# A Mobile System to Improve Quality of Life Via Energy Balance in Breast Cancer Survivors (BENECA mHealth): Prospective Test-Retest Quasiexperimental Feasibility Study

**DOI:** 10.2196/14136

**Published:** 2019-06-25

**Authors:** Mario Lozano-Lozano, Irene Cantarero-Villanueva, Lydia Martin-Martin, Noelia Galiano-Castillo, Maria-José Sanchez, Carolina Fernández-Lao, Paula Postigo-Martin, Manuel Arroyo-Morales

**Affiliations:** 1 Department of Physical Therapy Faculty of Health Sciences University of Granada Granada Spain; 2 Sport and Health University Research Institute Granada Spain; 3 Biohealth Research Institute in Granada Granada Spain; 4 Cuidate-Support Unit for Oncology Patients Granada Spain; 5 Andalusian School of Public Health Granada Spain; 6 Consortium for Biomedical Research in Epidemiology and Public Health Madrid Spain

**Keywords:** mHealth, energy balance, monitoring, breast cancer, survivors, quality of life

## Abstract

**Background:**

Energy balance is defined as the difference between energy expenditure and energy intake. The current state of knowledge supports the need to better integrate mechanistic approaches through effective studies of energy balance in the cancer population because of an observed significant lack of adherence to healthy lifestyle recommendations. To stimulate changes in breast cancer survivors’ lifestyles based on energy balance, our group developed the BENECA (Energy Balance on Cancer) mHealth app. BENECA has been previously validated as a reliable energy balance monitoring system.

**Objective:**

Based on our previous results, the goal of this study was to investigate the feasibility of BENECA mHealth in an ecological clinical setting with breast cancer survivors, by studying (1) its feasibility and (2) pretest-posttest differences with regard to breast cancer survivor lifestyles, quality of life (QoL), and physical activity (PA) motivation.

**Methods:**

Eighty breast cancer survivors diagnosed with stage I to IIIA and with a body mass index over 25 kg/m2 were enrolled in this prospective test-retest quasi-experimental study. Patients used BENECA mHealth for 8 weeks and were assessed at baseline and the postintervention period. Feasibility main outcomes included percentage of adoption, usage, and attrition; user app quality perception measured with the Mobile App Rating Scale (MARS); satisfaction with the Net Promoter Score (NPS); and barriers and facilitators of its use. Clinical main outcomes included measuring QoL with the European Organization for Research and Treatment of Cancer QoL Questionnaire Core 30 (EORT QLQ-C30), PA assessment with accelerometry, PA motivation measure with a Spanish self-efficacy scale for physical activity (EAF), and body composition with dual-energy x-ray absorptiometry. Statistical tests (using paired-sample t tests) and Kaplan-Meier survival curves were analyzed.

**Results:**

BENECA was considered feasible by the breast cancer survivors in terms of use (76%, 58/76), adoption (69%, 80/116), and satisfaction (positive NPS). The app quality score did not make it one of the best-rated apps (mean 3.71, SD 0.47 points out of 5). BENECA mHealth improved the QoL of participants (global health mean difference [MD] 12.83, 95% CI 8.95-16.71, P<.001), and EAF score (global MD 36.99, 95% CI 25.52-48.46, P<.001), daily moderate-to-vigorous PA (MD 7.38, 95% CI 0.39-14.37, P=.04), and reduced body weight (MD −1.42, 95% CI −1.97 to −0.87, P<.001).

**Conclusions:**

BENECA mHealth can be considered feasible in a real clinical context to promote behavioral changes in the lifestyles of breast cancer survivors, but it needs to be enhanced to improve user satisfaction with use and functionality. This study highlights the importance of the use of mobile apps based on energy balance and how the QoL of breast cancer survivors can be improved via monitoring.

## Introduction

There is a direct relationship between energy imbalance and an increased risk of not only multiple cancers but also cancer mortality, and a worsening of the effects of the disease [1-3]. Energy balance is defined as the difference between energy expenditure and energy intake [4]. Energy intake that exceeds energy expenditure is the main driver of weight gain; thus, balancing both helps weight maintenance [5].

A panel of experts from the International Agency for Research on Cancer and the World Cancer Research Fund agreed that 16 types of cancer are probably associated with one of the more relevant consequences of energy imbalance, excess fat accumulation in the body, making obesity the second leading cause of cancer worldwide [1,6]. Moreover, since the first decade of the 2000s, the scientific evidence on the benefits of physical activity (PA) in the quality of life (QoL) of cancer survivors (known as “oncological exercise”) has grown exponentially, generating dozens of systematic reviews, several international guidelines, and the recommendation to include programs of exercise in cancer survivors care [7]. Dietary and exercise interventions can alter the energy imbalance associated with cancer and potentially decrease the QoL of cancer survivorship [5]. However, the literature shows that despite strong evidence of this association, an insurmountable barrier prevails between “what needs to be done” and “what patients really do,” observing a significant lack of adherence to the preceding interventions [1].

In today’s progressively technical world, the use of mobile apps in smart devices has become the norm. In the same way, patients increasingly use therapeutic mobile apps related to some form of cancer treatment [8]. More than 2500 mobile apps are defined as apps related to cancer, but this relationship is peripheral or based on unproven claims, such as apps for yoga and naturopathy that claim to help prevent or even cure cancer [9]. In 2017, 15% of studies conducted worldwide were aimed at digital health, with 75% of these studies being conducted in the United States [9]. Recently, 539 apps were considered in a systematic review, which concluded that the effectiveness of most of them had not been validated scientifically [8,10]. Duman-Lubberding and colleagues [11] have developed Oncokompas, an eHealth app to facilitate access to supportive cancer care and monitor cancer patients’ QoL [12], specifically in the case of breast cancer [13]. Another study by Gietema and colleagues [14] assessed the feasibility of the Runkeeper app to improve the level of PA of cancer patients. They concluded that there is a need to increase research in the area. Different studies and meta-analyses of cancer patients show the benefits of mHealth, which include reducing fatigue or pain [15,16], distance PA programs with inconclusive results for and against [17-19], the use of social networks by patients of some types of cancer to improve QoL [20], and monitoring of symptoms [21,22]. However, none of these studies refers to monitoring and providing high-quality research feedback to restore the energy balance in cancer patients. The only references found in this field were in healthy populations [23,24], children and adolescents [25], pregnant women [26], hospitalized patients [27], and cardiac surgery [28] and diabetes [29] patients. Furthermore, monitoring using globally extended systems, such as Fitbit wristbands, is being questioned [30]. A recent systematic review of 67 studies concluded that, except for the measurement of steps in adults, there are a limited number of situations in which these devices provide accurate measurement for use in research [30].

In an attempt to stimulate changes in breast cancer survivors’ lifestyles based on energy balance, we developed the BENECA mHealth app: Energy Balance on Cancer [31,32]. BENECA mHealth aims to monitor the energy expenditure and energy intake of breast cancer survivors and provide instantaneous, simple, and clear feedback on the users’ energy balance, along with recommendations on how to improve it. This strategy was based on a recent systematic review of ﻿behavior change techniques for increasing PA in cancer survivors [33], as well as another study carried out by Hillier et al [34], who developed a Web-based program to assess energy balance in healthy adults. The first essential step, to develop and validate our tool, was to ensure the reliability of the BENECA mHealth monitoring system. The results of our previous study showed that it is a direct, rapid, and consistent evaluation system [32]. Based on these results, the goal of this study was to investigate the feasibility of BENECA mHealth in an ecological setting with a population of cancer survivors after they are discharged from their oncology treatment.

This involved studying the adoption of the app, its usage, user app quality perception, and the barriers and facilitators of its use. In addition, we gained insight into pretest-posttest differences with regard to breast cancer survivors’ lifestyles, QoL, and PA motivation. This investigation was based on the hypothesis that using the BENECA mHealth app for 8 weeks would help increase the motivation of breast cancer survivors to adhere to healthier lifestyles, thereby improving their QoL.

## Methods

### Study Design and Patient Recruitment

A prospective test-retest quasi-experimental study was carried out with 80 breast cancer survivors. The breast cancer survivors were selected based on the following eligibility criteria: (1) breast cancer stage I, II, or IIIA, (2) 30 to 75 years old, (3) body mass index (BMI) over 25 kg/m^2^, (4) user-level skills for app management, and (5) completed the adjuvant treatment at least 6 months before being included in the study. Eligible participants were excluded if they had mental or physical health conditions that prevented them from walking and/or participating in the assessment or if they did not sign the informed consent form. In addition, participants had to have access to a mobile device or tablet with an internet connection and an Android operating system. The research team loaned out two devices in cases where this was not possible or the operating system was incompatible with the app. All participants were recruited through the oncology unit from the University Hospital Complex of Granada, Spain, after being informed about the study and being referred by their respective oncologist. All eligible participants were contacted via telephone, screened using the inclusion and exclusion criteria, and if they were interested in participating, cited for the baseline assessment.

This study was approved by the ethics committee of the Andalusian Health Service (FIS, PI14-01627; Granada, Spain) ﻿and it was performed in accordance with the Helsinki Declaration for biomedical research (14/2017) [35]. Participants completed informed consent forms before the assessment.

### BENECA mHealth

The CUIDATE research group developed the Energy Balance on Cancer (BENECA) mHealth app to monitor and provide feedback to breast cancer survivors on healthy eating and PA. A description of the BENECA mHealth System [31,36] and a reliability study for the same [32] were previously published. After the baseline assessment was performed, a member of the research group downloaded the app on a patient’s mobile phone and taught them how to use it. The patient then had to prove that she understood the instructions by using the app in the presence of the researcher. Patients had to use BENECA mHealth for 8 weeks during the study. Physical activity (duration and intensity) and diet (food and drink intake) data were recorded via the app (self-recorded). Intake was recorded using a dietary record questionnaire; BENECA is structured with six consumption times. On each day, for each period, users report all food and beverages consumed. For PA, BENECA incorporated the Minnesota Leisure-time Physical Activity Questionnaire. Patients had to record intensity and duration of activities each day; BENECA only recorded those activities with a duration of at least 10 minutes. Using this information, the app sent a notification to the user of their daily energy balance, offering recommendations on diet and PA, which were based on the guidelines of the World Cancer Research Fund International, the strategies for PA and diet in patients with cancer from the American College of Sports Medicine [37], and the recommendations of the American Cancer Society [38]. Users receive a straightforward daily notification if there has been an energy imbalance; any difficulties in handling the app were resolved via calls and text messages between the researcher and patient (Multimedia Appendix 1). BENECA had been developed based on the theories of learning, Goal-Setting Theory, and Social Cognitive Theory to include techniques such as reinforcement, facilitation, self-monitoring, goal setting, feedback on performance, and reviewing goals, which have demonstrated to be promising in increasing PA in different populations [33,39]. A video tutorial was made available to the patients to review the use of the app.

### Outcome Measures

Patient demographic and clinical data were obtained at the beginning of the study using a study-specific survey. Baseline data were gathered at the start of the study and again after 8 weeks of using BENECA mHealth. The outcomes measured are presented subsequently.

#### Feasibility of Main Outcomes

BENECA mHealth was considered feasible for use by breast cancer survivors as long as it met the following criteria, established based on previous studies with eHealth apps [11,13,40,41]: adoption and usage rate over 50%, a positive Net Promoter Score (NPS), and a Mobile App Rating Scale (MARS) score of up to 3.73 out of 5.

#### Adoption, Usage, and Attrition

The adoption rate was the percentage of the number of breast cancer survivors that agreed to participate in the study and completed the initial assessment, demonstrating the intention to use BENECA mHealth, out of the total number invited to participate in the study. The usage rate is the percentage of breast cancer survivors that used BENECA mHealth, which was determined through the logging data of the app. Both the adoption and usage rates were calculated based on the methods used in a previously published study [13]. The attrition rate is the percentage of breast cancer survivors that stopped using BENECA mHealth and did not use it again, as per Eysenbach’s definition [42]. To assess the safety of the process, any adverse effects reported by the patients were recorded through a patient’s daily diary.

#### BENECA mHealth Quality

The MARS was used to assess the quality of BENECA mHealth. The MARS is composed of 23 items grouped into different sections: engagement, functionality, aesthetics, and information quality (with which the overall average score of the scale is obtained). There are also two optional sections: subjective quality (with four items) and app-specific quality (with six items). Each item was assessed independently based on a Likert scale from 1 (inadequate) to 5 (excellent), and the mean score was calculated for each section. This scale has been validated and has proven to be simple, objective, and reliable to assess the quality of mHealth apps [43]. Similarly, the NPS was used to measure satisfaction based on responses to the following question: How likely are you to recommend BENECA mHealth to other breast cancer survivors? The responses were recorded using an 11-point Likert scale in which 0 indicates “not likely” and 10 indicates “very likely.” The percentage of *detractors* (those whose scores were from 0 to 6) and *promoters* (those whose scores were from 9 to 10) were calculated, and each group was given a score between −100 and 100. A positive score is considered good; a negative score is considered bad [44]. This methodology has been used as a predictor of growth and an indicator of customer satisfaction in for-profit industries, and it provides insight into the client experience in nonprofit health care settings [45].

#### Barriers and Facilitators

After the participants used BENECA mHealth for 8 weeks and completed the corresponding assessment, a trained member of the research team interviewed each participant using a standardized set of interview questions based on a previous study [13]. This interview focused on three main elements: overall experience with BENECA mHealth, congruence between expectations and reality with BENECA mHealth, and the perception and added value of BENECA mHealth. For cases in which the app was no longer used, the participants were asked about their reasons for not using the app and the preferences or needs that would prompt them to use it. Each interview was read several times and transcribed by the same researcher, and the barriers and facilitators reported by the breast cancer survivors were synthesized [46].

### Main Clinical Outcomes

#### Quality of Life

The European Organization for Research and Treatment of Cancer QoL Questionnaire Core 30 (EORTC QLQ-C30) version 3.0 was used to assess the QoL of the participants. This questionnaire is intended to measure general aspects of QoL specific to cancer patients. It contains five functional scales (physical, role, cognitive, emotional, and social functioning), a global health status scale, and symptom scales of fatigue, nausea and vomiting, pain, dyspnea, insomnia, appetite loss, constipation, diarrhea, and financial problems. It is scored using a four-point Likert scale (from 1=“not at all” to 4=“very much”) and the raw scores are transformed into a 0 to 100 scale. The higher the score on the functional scales, the better the QoL, but the higher the score on the symptom scales, the poorer the QoL [47,48].

#### Self-Efficacy and Motivation in Relation to Physical Activity

A Spanish self-efficacy scale for physical activity (EAF) was used to measure the self-efficacy and motivation of the participants to engage in PA and incorporate it into their daily activities. It consists of three domains: scheduled physical exercise, PA in daily life activities, and walking, which determine a person’s perception of their abilities to engage in PA (self-efficacy for PA) [49].

#### Physical Activity

Data on PA and the sedentary lifestyle of the breast cancer survivors were collected using accelerometry based on a previously published protocol of use and analysis [50]. A preprogrammed triaxial accelerometer (ActiGraph GT3X+, Pensacola, FL, USA) was used by each patient for eight consecutive days. The participants received a questionnaire diary and an instruction sheet on how to use the device. Only the records of more than 4 days and of at least 10 hours per day were included in the analysis.

#### Body Composition

Dual-energy x-ray absorptiometry (Discovery DXA densitometer from Hologic, QDR 4500 W) was used for assessing BMI, the percentage of fat mass, and bone mineral density, as previously used for breast cancer patients [51] in accordance with protocol of use [52]. The height and weight of the participants were also measured at baseline as well as hip and waist circumferences.

### Statistical Analysis

All analyses were performed using SPSS Statistics version 24 (IBM Corp, Armonk, NY, USA). Statistical significance was assumed when *P*<.05. The logging data from BENECA mHealth were obtained on request from the computer engineers responsible for the development of the app.

First, descriptive measures were used to report the data on adoption, use, attrition, and quality, as well as to report on the clinical and anthropometric variables of the participants. A Kaplan-Meier survival curve was used to visually examine the survival curve of the entire cohort to determine the attrition. In the analysis, an “app survivor” was defined as a breast cancer survivor that maintained logging practices using BENECA mHealth until at least 3 days before the last day of the experimental period. Those defined under “app death” were those who missed five consecutive daily loggings (based on a previous study [53]). A Kaplan-Meier estimator with right-censored data was used. This type of data was used because it best fit our study results. As most of the breast cancer survivors “survived” until the end of the experimental period, we do not know how long they would have continued using BENECA mHealth after this period. Then, a Cox proportional hazard model was used to examine if age, marital status, and employment had any effect on the attrition.

Second, to assess the pretest-posttest differences in the main outcomes, an analysis of paired-sample *t* tests was used and, when appropriate, Wilcoxon signed rank tests were conducted. Moreover, the effect size (ES) estimate was determined and interpreted using Cohen’s guidelines of 0.1=small effect, 0.3=medium effect, and 0.5=large effect.

Third, to assess differences between “users” and “nonusers” and the patients’ perception of BENECA mHealth quality, a Mann-Whitney test was used for categorizing the breast cancer survivors according to the cut-off used in the survival analysis. A simple linear regression was used to examine the influence of age on the perception of BENECA mHealth quality.

Our data contained a few missing values (5%, 4/80 of the total number of cases), but these can be considered random and inconsequential [54]. Hence, no multiple imputation method was necessary (casewise deletion was used).

## Results

### Demographic Characteristics

The baseline demographic and clinical characteristics of the participants (mean age 51.80, SD 8.64 years) are presented in Table 1. Of the 80 breast cancer survivors, 50 (62%) were married, 31 (38%) had a higher education, and 40 (50%) were diagnosed with stage II breast cancer, followed in frequency by stage IIIA (28/80, 35%). All participants received instructions on how to use BENECA mHealth to monitor energy intake and expenditure. Four participants were unable to be assessed postintervention (dropouts); three were not assessed due to changes in their health status unrelated to the study, and one decided to discontinue.

**Table 1:** Participant demographics (N=80).

Variables		Participants
Age (years), mean (SD)		51.80 (8.64)
Body mass index, mean (SD)		29.11 (4.77)
**Marital status, n (%)**		
	Single	16 (20)
	Married	50 (63)
	Divorced	10 (13)
	Other	4 (5)
**Education, n (%)**		
	No education	1 (1)
	Primary studies	23 (29)
	Secondary studies	25 (31)
	Higher education	31 (39)
**Employment, n (%)**		
	Homemaker	18 (22)
	Employee	32 (40)
	Low	10 (13)
	Unemployed by the disease	20 (25)
**Cancer stage, n (%)**		
	I	10 (13)
	II	40 (51)
	IIIA	28 (36)
**Surgery, n (%)**		
	Lumpectomy	24 (30)
	Quadrantectomy	13 (16)
	Unilateral mastectomy	27 (34)
	Bilateral mastectomy	16 (20)
**Medical treatment, n (%)**		
	None	6 (8)
	Radiation therapy alone	10 (13)
	Chemotherapy alone	6 (8)
	Chemotherapy and radiation therapy	48 (60)
	Adjuvant chemotherapy	7 (9)
	Neoadjuvant chemotherapy	3 (4)

### Feasibility Outcomes

#### Adoption, Usage, and Attrition Rates

**Figure 1:**
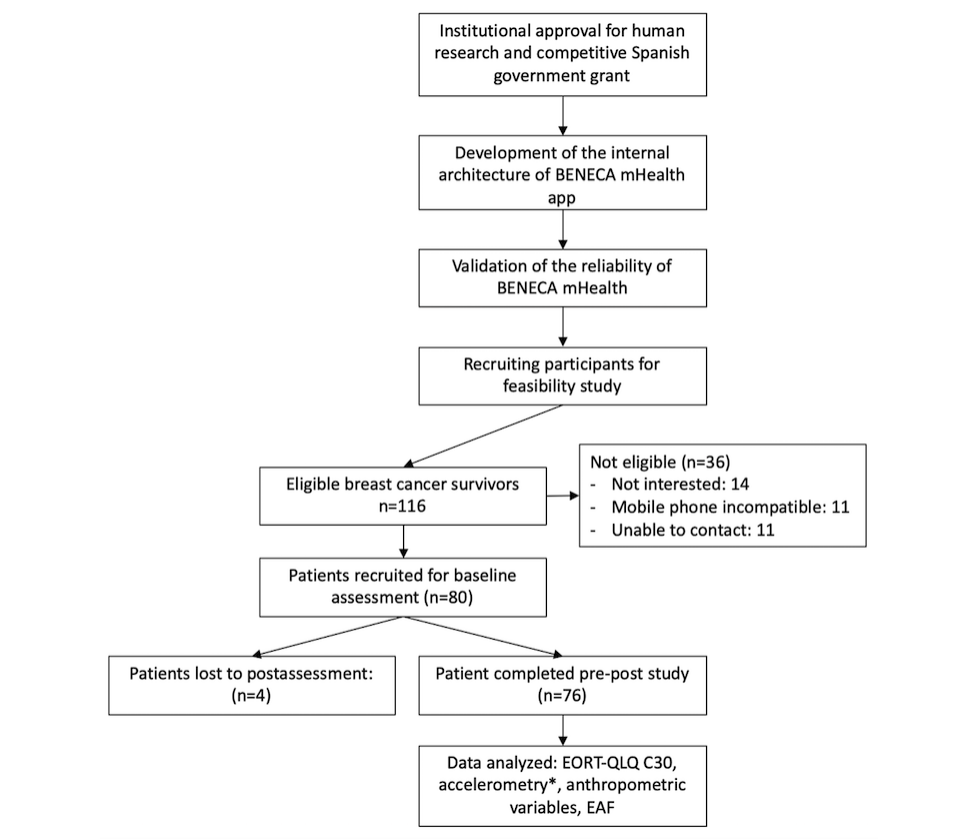
Flow diagram of the study design. EAF: self-efficacy scale for physical activity; EORT QLQ-C30: European Organization for Research and Treatment of Cancer Quality of Life Core Questionnaire 30. *N=75 for accelerometry analyses (one broken device on preassessment).

The study design is shown in Figure 1. The adoption rate of BENECA mHealth was 69%; 80 of 116 breast cancer survivors who were invited to participate intended to use BENECA mHealth, filled the informed consent form, and were assessed at baseline. The reasons for not participating in the study included lack of interest (too busy; n=14), incompatibility of the user’s mobile operating system with BENECA mHealth (n=11), and failed initial contact (eg, wrong phone number or no answer; n=11).

**Figure 2:**
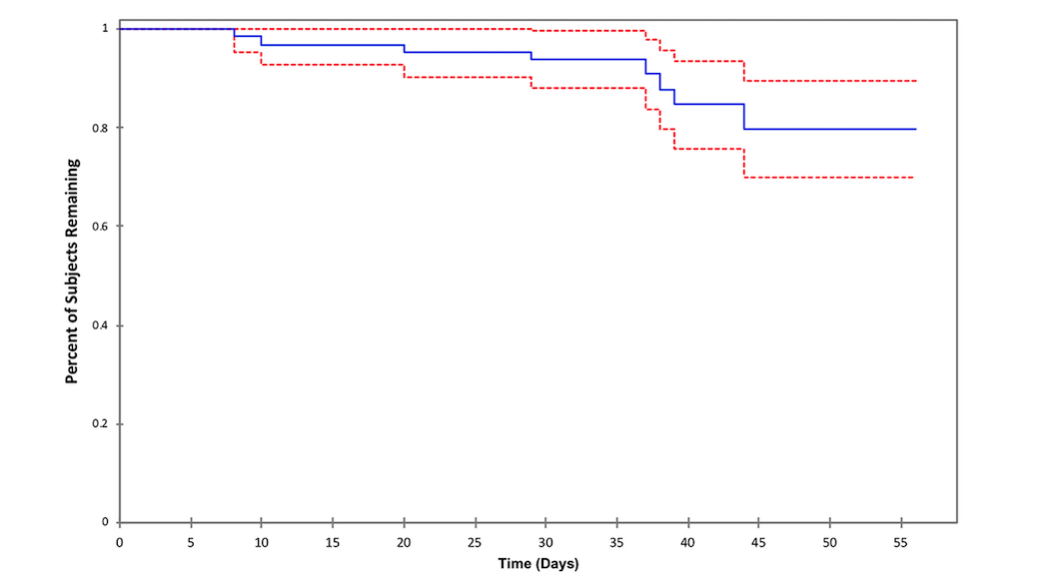
“Survival” of BENECA app participants as shown by a Kaplan-Meier survival curve with 95% CIs (dashed lines).

The usage rate was 73% (58/80) including dropouts and 76% (58/76) excluding dropouts. The reasons for stopping using BENECA mHealth included technical issues, such as difficulty in finding specific foods (n=6), app blocks (n=4), difficulty in calculating proportions of diet registration (n=9), or lack of motivation (n=3). We examined attrition using the Kaplan-Meier survival curve and Cox proportional hazards model. Figure 2 illustrates the attrition curve of the study participants with their respective 95% CIs. The curve is flat at the beginning, begins to get steeper after the first month, and flattens again with time. The Cox proportional hazards model was used to assess the differences in the survival rate using covariables that could affect this rate from the clinical point of view based on a priori knowledge. The results obtained using this model with the covariates were significant at *P*=.02; the coefficients are shown in Table 2.

**Table 2:** Coefficients for the Cox proportional hazards model.

Coefficients		Coefficient estimate (95% CI)	*P* value
Age		1.12 (1.04-1.19)	.001
**Marital status^a^**			
	Married	0.88 (0.25-3.18)	.85
	Divorced	0.77 (0.15-4.04)	.76
	Other	2.52 (0.35-18.26)	.36
**Employment^b^**			
	Employee	0.46 (0.13-1.59)	.22
	Low^c^	1.12 (0.27-4.62)	.87
	Unemployed due to the disease	0.46 (0.12-1.67)	.24

^a^Marital status reference category: single.

^b^Employment reference category: homemaker.

^c^Unemployed/on leave.

### Patients’ Perception of BENECA mHealth Quality

**Figure 3:**
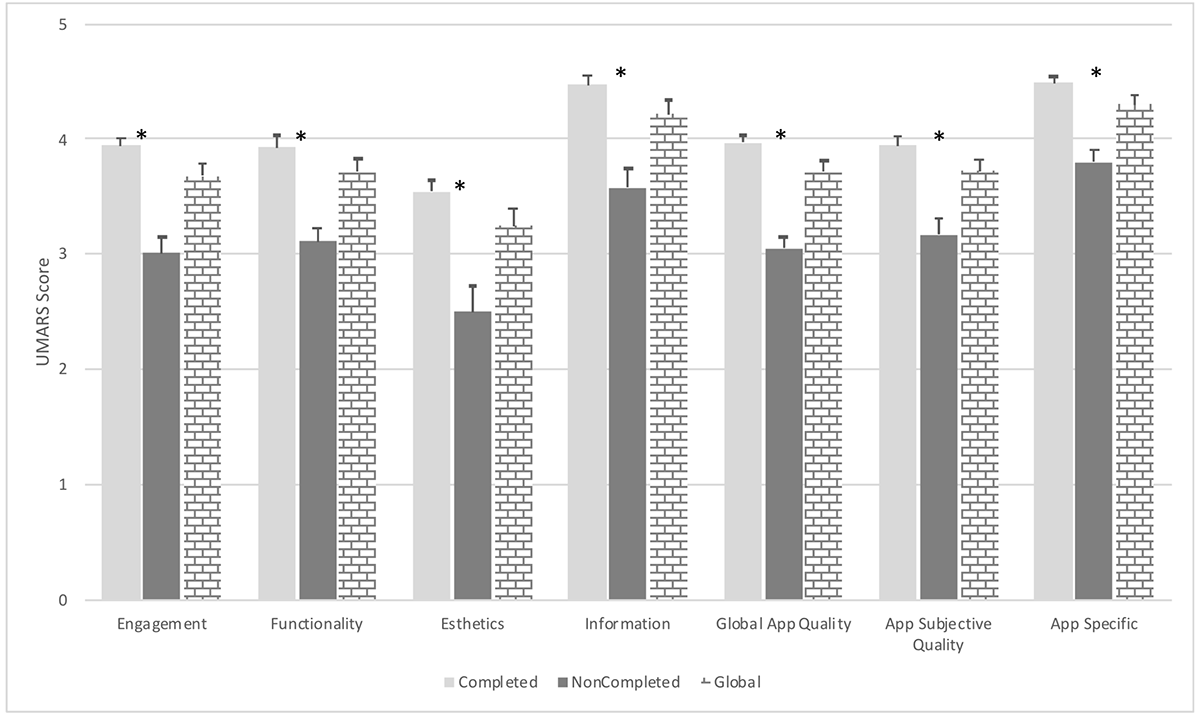
Mobile App Rating Scale (MARS) mean scoring. Data show differences between completed and noncompleted users and global mean scoring. Completed users are defined as those who used the BENECA mHealth app until study completion (n=58). Noncompleted users are defined as those who stopped using the BENECA mHealth app study completion (n=22). *P<.001. UMARS: User Version of the Mobile Application Rating Scale.

The mean MARS quality score for the app was 3.71 (SD 0.47) out of 5, and the NPS was positive (6.58 in range of −100 to 100), consisting of 24% (19/80) detractors, 30% (24/80) promoters, and 46% (37/80) passives. On average, the best-rated MARS category was app-specific change (mean 4.30, SD 0.37), followed by information (mean 4.22, SD 0.51), app subjective quality (mean 3.73, SD 0.46), and functionality (mean 3.71, SD 0.52). The worst-rated section was aesthetics, with a mean of 3.25 (SD 0.63). The specific scores for each section of the MARS are illustrated in Figure 3. The participants were divided according to the cut-off used in the survival analysis. It shows how the participants who used BENECA until the end of the experimental period scored higher and were statistically significant in all sections (*P*<.001). Linear regression showed that the older the patient, the lower the app quality score (beta=−0.29, *t*_75_=−2.64, *P*=.01).

### Barriers and Facilitators

In summary, seven barriers and five facilitators were quoted five times or more when the participants were interviewed. Among the barriers, the most common was “BENECA does not have some food items” followed by “difficulty at the time of introducing the intake.” Among the facilitators, the most common was “BENECA provides relevant information to the patient” followed by “patient considers it important to know BENECA’s feedback on energy balance.” Table 3 summarizes the barriers and facilitators mentioned.

**Table 3:** Barriers and facilitators toward the feasibility of the BENECA mHealth app (N=77).

Barriers and facilitators		n (%)
**Barriers**		
	Extension of BENECA	29 (38)
	BENECA does not have an added value for the patient	9 (12)
	BENECA does not have some food items	59 (77)
	BENECA does not have some physical activities	8 (10)
	BENECA feedback is limited	17 (22)
	Difficulty at the time of introducing the intake	42 (55)
	The patient’s perception of BENECA’s contribution to her health is negative	2 (3)
**Facilitators**		
	The usefulness of BENECA in general	32 (42)
	Ease of introducing physical activity	27 (35)
	Patient considers it important to know BENECA’s feedback on energy balance	55 (71)
	BENECA is easy to use	18 (23)
	BENECA provides relevant information to the patient	51 (66)

### Main Clinical Outcomes

#### Quality of Life

The results of the main pre-post analyses of EORT QoL C30 are shown in Table 4. Statistically significant differences were observed after the experimental period with moderate to large effects as follows: general QoL (*t*_75_=6.592, *P*<.001, *d*=0.87), physical functioning (*t*_75_=5.312, *P*<.001, *d*=0.63), emotional functioning (*t*_75_=2.981, *P*=.004, *d*=0.23), cognitive functioning (*t*_75_=5.575, *P*<.001, *d*=0.75), social functioning (*t*_75_=6.619, *P*<.001, *d*=0.82), fatigue (*t*_75_=−6.003, *P*<.001, *d*=0.85), pain (*t*_75_=−2.017, *P*=.047, *d*=0.23), dyspnea (*t*_75_=−5.190, *P*<.001, *d*=0.61), and insomnia (*t*_75_=−2.905, *P*=.005, *d*=0.32). An improvement in the scores of all these items, as well as a reduction in some symptoms, was observed after 2 months of using BENECA mHealth.

**Table 4:** Within-group pre-post effects on mean quality of life scores on the European Organization for Research and Treatment of Cancer Quality of Life Core Questionnaire 30 (EORTC QLQ-C30).

EORT QLQ-C30 variable	Study group, mean (SD)		Mean difference (95% CI)	*P* value^a^
	Pre (n=76)	Post (n=76)		
Global health	58.54 (14.40)	70.83 (11.26)	12.83 (8.95 to 16.71)	<.001
Physical functioning	75.25 (15.88)	85.35 (13.16)	10.88 (6.80 to 14.96)	<.001
Role functioning	66.45 (26.45)	70.83 (24.36)	5.26 (−1.99 to 12.52)	.15
Emotional functioning	59.06 (19.31)	64.04 (19.82)	5.59 (1.86 to 9.33)	.004
Cognitive functioning	62.5 (22.11)	80.26 (21.38)	17.98 (11.56 to 24.41)	<.001
Social functioning	66.88 (23.94)	86.62 (20.00)	20.17 (14.10 to 26.25)	<.001
Fatigue	42.5 (23.64)	23.68 (15.95)	−19.59 (−26.09 to −13.09)	<.001
Nausea	2.29 (6.35)	2.19 (5.67)	−0.22 (−1.54 to 1.10)	.95
Pain	44.58 (26.22)	38.6 (20.59)	−6.35 (−12.64 to −0.08)	.047
Dyspnea	27.92 (25.13)	12.72 (19.60)	−15.35 (−21.24 to −9.46)	<.001
Insomnia	46.25 (36.16)	35.09 (32.61)	−12.28 (−20.70 to −3.86)	.005
Appetite loss	9.58 (15.18)	7.46 (15.00)	−2.19 (−6.38 to 1.99)	.30
Constipation	21.67 (28.11)	19.74 (29.40)	−1.75 (−8.78 to 5.27)	.62
Diarrhea	10.83 (19.68)	12.72 (18.83)	1.31 (−1.85 to 4.47)	.41
Financial difficulties	19.17 (28.94)	16.67 (24.65)	−2.19 (−5.99 to 1.61)	.25

^a^Paired-sample *t* test or Wilcoxon signed rank test as appropriate. Analyses were performed on only those patients that followed-up.

#### Self-Efficacy and Motivation for Physical Activity and Accelerometry

The results of the main pre-post analyses using the self-efficacy scale for PA and accelerometry are shown in Table 5. There were significant statistical differences after the experimental period with a moderate ES on the EAF scale as follows: daily PA (*t*_75_=5.369, *P*<.001, *d*=0.56), walking (*t*_75_=6.228, *P*<.001, *d*=0.55), and total EAF score (*t*_75_=6.423, *P*<.001, *d*=0.67). For accelerometry, there were only significant differences in weekday moderate-to-vigorous physical activity (MVPA; *t*_75_=2.106, *P*=.04, *d*=0.26), observing trend in global MVPA (*t*_75_=1.917, *P*=.06), weekday steps (*t*_75_=1.779, *P*=.08), and global steps (*t*_75_=1.647, *P*=.10). Therefore, after using BENECA mHealth, the users felt more motivated to increase the levels of PA in their daily lives.

**Table 5:** Within-group pre-post effects on mean scores on the self-efficacy scale for physical activity (EAF) and accelerometry.

Variable		Study group, mean (SD)		Mean difference (95% CI)	*P* value^a^
		Pre (n=76^b^)	Post (n=76)		
**EAF**					
	Scheduled PA^c^	81.70 (33.08)	87.71 (19.22)	6.08 (−1.08, 13.24)	.10
	Daily live PA	50.06 (22.67)	62.63 (17.64)	12.22 (7.69, 16.76)	<.001
	Walking	15.20 (9.03)	20.34 (7.95)	5.12 (3.48, 6.76)	<.001
	Total EAF score	146.96 (53.36)	184.61 (48.52)	36.99 (25.52, 48.46)	<.001
**Accelerometry**					
	MVPA^d^ weekday	50.68 (25.83)	58.07 (26.05)	7.38 (0.39, 14.37)	.04
	MVPA weekend	41.77 (24.55)	42.77 (21.51)	0.99 (−4.62, 6.62)	.73
	MVPA global	48.14 (24.31)	53.69 (21.85)	5.55 (−0.22, 11.34)	.06
	Steps weekday	7488.97 (3142.34)	8268.41 (3230.87)	779.44 (−94.35, 1653.22)	.08
	Steps weekend	6218.50 (3147.26)	6316.87 (2875.87)	98.37 (−678.14, 874.88)	.80
	Steps global	7125.97 (2935.94)	7710.82 (2672.78)	584.85 (−123.09, 1292.78)	.10

^a^Paired-sample *t* test or Wilcoxon signed rank test as appropriate. Analyses were performed on only those patients that followed-up.

^b^Accelerometry analyses was perform on 75 participants because there was one more dropout on preassessment (broken device).

^c^PA: physical activity.

^d^MVPA: moderate-to-vigorous physical activity.

#### Body Composition

The results of the main pre-post analyses of the anthropometric variables are shown in Table 6. Statistically significant differences were observed after the experimental period with a moderate ES as follows: weight (*t*_75_=−5.050, *P*<.001, *d*=0.12) and BMI (*t*_75_=−4.804, *P*<.001, *d*=0.12). In addition, a trend was observed in waist circumference (*t*_74_=−1.900, *P*=.06) and body fat (*t*_75_=−1.946, *P*=.06). No differences were observed for hip circumference (*t*_74_=−1.007, *P*=.32) and bone mineral density (*t*_75_=−1.019, *P*=.31). After 2 months of using BENECA mHealth, a reduction in users’ body weight was observed, which could lead to a reduction in the hip circumference and percentage of body fat.

**Table 6:** Within-group pre-post differences on anthropometric and body composition variables.

Variable	Study group, mean (SD)		Mean difference (95% CI)	*P* value^a^
	Pre (n=76)	Post (n=76)		
Weight (kg)	73.09 (11.14)	71.67 (10.90)	−1.42 (−1.97, −0.86)	<.001
BMI^b^ (kg/m^2^)	29.11 (4.78)	28.51 (4.73)	−0.57 (−0.81, −0.34)	<.001
Waist circumference (cm)	87.45 (9.26)	86.97 (9.00)	−0.84 (−1.71, 0.04)	.06
Hip circumference (cm)	107.94 (14.23)	107.71 (13.11)	−0.64 (−1.93, 0.63)	.32
Body fat (%)	41.44 (6.23)	39.78 (7.34)	−1.57 (−3.18, 0.04)	.06
Bone mineral density (g/cm^2^)	1.02 (0.11)	1.04 (0.14)	0.02 (−0.02, 0.05)	.31

^a^Paired-sample *t* test or Wilcoxon signed rank test as appropriate. Analyses were performed on only those patients that followed-up.

^b^BMI: body mass index.

## Discussion

### Principal Results

According to our initial hypothesis, after using BENECA mHealth for 8 weeks, the app was considered feasible by the breast cancer survivors in terms of use, adoption, and satisfaction, although the app quality score did not make it one of the best-rated apps. BENECA mHealth was associated with changes in the QoL of breast cancer survivors, as well as their self-perception of effectiveness and motivation for engaging in PA in their daily life.

### Comparison With Prior Work

The adoption rate in this study was 69%, and the usage rate was 73% to 76%. These results can be explained by the technical characteristics of BENECA mHealth and its functionality, such as user-friendliness, the use of internationally accepted measures, and the visual feedback. The results of this study are comparable with those obtained by Melissant et al [13] for a supportive care app for breast cancer survivor, which had an adoption rate of 75% and usage rate of 75% to 84%. Another study of a lifestyle intervention with a mobile app for endometrial and breast cancer survivors recorded a 75% usage rate [55]. However, Duman-Lubberding et al [11] obtained an adoption rate of 64% and a usage rate of 75% to 91% for a similar app for head and neck cancer survivors. The somewhat lower rate of use in our study for the latter may be due to how these data were obtained (ie, by the number of log-ins—objective measure—instead of the self-reported data of those studies—subjective measure). With regard to “app survival,” we found that in a study by Springer et al [53] to test an mHealth app targeting healthy eating behavior in the general population, they obtained a survival rate less than 60% using the Kaplan-Meier survival curve. The higher survival rate in our study (over 70%) can be explained by the type of population studied. In general, patients with some type of pathology will be more predisposed to be involved in this type of study than the general population [56]. In addition, experiencing cancer treatment may be a stimulus to use the app, as patients may feel the increased need to learn more about the treatment.

Taking into account the barriers perceived by the participants in the use of the app, the barriers reported by BENECA mHealth were in line with a recently published review on the adherence to online psychological interventions [57] as well as with those in a study by Melissant et al [13] with the Oncokompas app to monitor the QoL of breast cancer survivors (eg, “Oncokompas is too extensive”). The reported mean satisfaction score of the quality of BENECA mHealth, although it may seem not very high, is in line with a recently published study on the quality of 18 mobile apps for pain management using the same MARS quality scale [41]. In addition, the low scores in some sections can be explained by the barriers reported by the patients, such as the difficulties in inputting the intake that makes it very extensive to fill in the app. This barrier was also reported in another feasibility study on head and neck cancer patients [11]. Considering that the minimum score to be considered a best-rated app based on the MARS scale is 3.73 (according to a previous study [41]), BENECA mHealth can be regarded as an app with average ratings. BENECA is currently being improved in an attempt to address the reported barriers.

The benefits of PA for cancer patients have been amply demonstrated [58], although a recent meta-analysis (2013-2018) of distance-based PA behavioral change interventions for cancer survivors concluded that the effects of interventions on PA were small [18]. In addition, although efficacy cannot be discussed in a study such as this, according to the literature, a difference of 8 points between assessments of QoL measured with the EORT QLQ-C30 is the minimum clinically significant difference required to discuss the clinical relevance of the findings [59]. The QoL findings in this study reinforce these preceding conclusions and are consistent with the results of the EAF scale and those observed via accelerometry. Changes are observed in the participants with more motivation to do PA, and it seems that using BENECA mHealth is associated with changes that lead to a positive feedback chain that improves physical and emotional functioning. The significant differences in cognitive functioning can be explained by the actual use of the mobile device, as there is evidence of the cognitive benefits of using electronic devices [60]. Our findings are in agreement with those reported by Pope et al [20], who used a mobile app and social media for 10 weeks to improve the QoL of breast cancer survivors, with a sample size much smaller than ours. However, they differ from the conclusions of McCarroll et al [55], who assessed the effectiveness of a public mobile app (LoseIT) for dietetic intervention for 4 weeks in breast and endometrial cancer survivors. They did not find significant changes in the QoL of the patients. It is possible that the experimental period of 4 weeks and lack of stratification of the type of cancer could explain these differences, despite the use of a powerful questionnaire to assess QoL. Lastly, we only found statistically significant differences in the MVPA of the data obtained via accelerometry, although we observed an improvement in other variables after the use of BENECA mHealth. These results are consistent with those of a clinical trial published in 2018 that used smartwatches and social media PA behavioral change over a 10-week intervention to determine the health outcomes for breast cancer survivors, in which no significant differences in the accelerometry variables were observed [61].

Finally, one of the main challenges not only with cancer patients but with the general population is the maintenance and reduction of body weight [5,62]. Different studies of lifestyle interventions have shown beneficial results, such as the one by von Gurenigen et al [63] in which they evaluated the effectiveness of a face-to-face intervention on diets in obese patients with endometrial cancer, achieving a reduction of approximately 5%. Similarly, McCarroll et al [55] achieved a reduction of approximately 6% from baseline weight. The literature indicates that a weight reduction of 5% is sufficient to reduce medical comorbidities [62]. In our study, an average weight loss of approximately 2% was achieved, which is below the recommendations. This may be because BENECA mHealth is not really a lifestyle intervention mobile app, but rather one that tries to incite behavioral change in users by monitoring their energy balance and making them aware of it. Therefore, we believe that the results obtained can be considered a first step, although future research should corroborate these results. The internal architecture of BENECA mHealth can also be extrapolated to suit patients with other types of cancer.

### Strengths and Limitations

It is important to recognize some of the limitations of this study. The main one is its design. It is a nonrandomized, single-arm exploratory study; therefore, the results should be taken with caution. The ideal study would have been a randomized controlled trial (RCT); nevertheless, it was mandatory to develop a feasibility study for this sensitive population before carrying out an RCT. Moreover, due to the nature of the design of this study, the reported results must be confirmed in a larger RCT because the observed changes may not be attributable to the intervention. Secondly, BENECA was only developed for the Android operating system, but we are currently working on the next version of the BENECA app to solve this limitation. Thirdly, BENECA was designed to monitor energy balance and then propose recommendations based on international guidelines of clinical practice, systematic reviews, and meta-analysis to ensure the recommendations can be generalized. However, we believe that it is a good starting point, especially for very sedentary people. Finally, the generalization of results is limited due to the design of the study, the use of restrictive inclusion and exclusion criteria and the recruitment strategy (the participants were referred by their oncologists), which may involve a bias of the threat of regression to the mean. In addition, another added difficulty could refer to the use of the app by older people in southern Spain, who may not even have mobile phones adapted to the app. Therefore, future studies should be conducted with a larger sample size; a controlled and randomized clinical trial design comparing the use of BENECA with, for example, a face-to-face intervention; and including biomarker measurements such as those for inflammation or development/recurrence of breast cancer.

Despite these limitations, this study also has strengths. These include the wide range of ages of the participants, which makes it possible to generalize the results; the use of energy balance as a means of changing behavior, which has not been studied much; its ease of use; it has high adherence; and it has no adverse effect on the prior validation of BENECA mHealth [32], which guarantees its reliability.

### Conclusions

BENECA mHealth can be considered feasible in a real clinical context and has been associated with behavioral changes in the lifestyles of breast cancer survivors, but it needs to be enhanced to improve user satisfaction with use and functionality. Having assumed that BENECA is usable and applicable in a real clinical context, as well as having the first data of its applicability and clinical efficacy, the next step will be to confirm these results through a larger study with a control group. In addition, efforts should focus on overcoming the barriers reported by the participants and developing a new version of BENECA mHealth in which these improvements will be implemented. Finally, future research could focus on its generalization for application to other oncological processes. This study highlights the importance of the use of mobile apps based on energy balance and how the QoL of breast cancer survivors can be improved via monitoring. The results of this study could garner support for the use of this type of strategy in the projected 29.5 million cancer patients in 2040 [64].

## Multimedia Appendix 1

Examples of feedback messages of the BENECA mHealth app.

## Abbreviations

BMI: body mass index
EAF: self-efficacy scale for physical activity (Spanish)
EORT QLQ-C30: European Organization for Research and Treatment of Cancer Quality of Life Questionnaire Core 30
ES: effect size
MARS: Mobile App Rating Scale
MVPA: moderate-to-vigorous physical activity
NPS: Net Promoter Score
PA: physical activity
QoL: quality of life
RCT: randomized controlled trial
