# Supplementary material for: A Mobile System to Improve Quality of Life Via Energy Balance in Breast Cancer Survivors (BENECA mHealth): Prospective Test-Retest Quasiexperimental Feasibility Study
Source: JMIR Mhealth Uhealth. 2019 Jun 25;7(6):e14136. doi: 10.2196/14136 (PMC6614997; doi:10.2196/14136)
Supplement: Supplementary file 1 [file mhealth_v7i6e14136_app1.pdf]

Examples of feedback messages of BENECA mHealth:

1. Daily reminders of intake register, in each period of intake.
2. Daily reminders of physical activity register, at the end of the day.
3. Notification of daily energy balance.
4. Weekly achievements (messages related to):
    a. Physical activity.
    b. Energy balance
5. Weekly, consecutive achievements achieved.
6. 6. Notification of "no activity", if in a couple of days, or a week, the application is not used, notification of "we miss you".
7. Notifications of "update your anthropometric data", weight, circumferences ...
